# Downregulation of lysyl oxidase and lysyl oxidase-like protein 2 suppressed the migration and invasion of trophoblasts by activating the TGF-β/collagen pathway in preeclampsia

**DOI:** 10.1038/s12276-019-0211-9

**Published:** 2019-02-21

**Authors:** Xiang-Hong Xu, Yuanhui Jia, Xinyao Zhou, Dandan Xie, Xiaojie Huang, Linyan Jia, Qian Zhou, Qingliang Zheng, Xiangyu Zhou, Kai Wang, Li-Ping Jin

**Affiliations:** Clinical and Translational Research Center, Shanghai First Maternity and Infant Hospital, Tongji University School of Medicine, 2699 West Gaoke Road, Shanghai, 201204 P. R. China

**Keywords:** Pre-eclampsia, Inflammation

## Abstract

Preeclampsia is a pregnancy-specific disorder that is a major cause of maternal and fetal morbidity and mortality with a prevalence of 6–8% of pregnancies. Although impaired trophoblast invasion in early pregnancy is known to be closely associated with preeclampsia, the underlying mechanisms remain elusive. Here we revealed that lysyl oxidase (*LOX*) and LOX-like protein 2 (*LOXL2*) play a critical role in preeclampsia. Our results demonstrated that *LOX* and *LOXL2* expression decreased in preeclamptic placentas. Moreover, knockdown of *LOX* or *LOXL2* suppressed trophoblast cell migration and invasion. Mechanistically, collagen production was induced in *LOX*- or *LOXL2*-downregulated trophoblast cells through activation of the TGF-β1/Smad3 pathway. Notably, inhibition of the TGF-β1/Smad3 pathway could rescue the defects caused by *LOX* or *LOXL2* knockdown, thereby underlining the significance of the TGF-β1/Smad3 pathway downstream of LOX and LOXL2 in trophoblast cells. Additionally, induced collagen production and activated TGF-β1/Smad3 were observed in clinical samples from preeclamptic placentas. Collectively, our study suggests that the downregulation of *LOX* and *LOXL2* leading to reduced trophoblast cell migration and invasion through activation of the TGF-β1/Smad3/collagen pathway is relevant to preeclampsia. Thus, we proposed that *LOX*, *LOXL2*, and the TGF-β1/Smad3/collagen pathway can serve as potential markers and targets for clinical diagnosis and therapy for preeclampsia.

## Preeclampsia: when cells aren’t invasive enough

Cancer-associated proteins play a role in preeclampsia, a potentially life-threatening disorder of pregnancy marked by high blood pressure and protein in the urine. The causes of preeclampsia are poorly understood, but the tissue that nourishes the fetus, the placenta, is known to be involved. Knowing that for healthy placenta formation, cells called trophoblasts must show cancer cell-like behavior and invade the developing tissue, Li-Ping Jin and Kai Wang at Tongji University School of Medicine in Shanghai, China, and co-workers investigated the role of cancer-associated LOX proteins, previously linked to cell invasiveness. Preeclamptic placentas showed low LOX levels, poor trophoblast invasion, and excessive formation of collagen, an important connective tissue. Further analysis showed that this excessive collagen is broken down, becoming the diagnostic urinary protein. These results illuminate potential markers for early diagnosis and treatment of preeclampsia.

## Introduction

The lysyl oxidase (LOX) protein family is comprised of five closely related members, prototypical LOX and four LOX-like proteins (LOXL1, LOXL2, LOXL3, and LOLX4)^1^. LOX proteins are primarily known for their roles as extracellular enzymes. Upon secretion, they catalyze the oxidative deamination of peptidyl lysine residues, promoting the formation of lysyl-derived crosslinking of collagen and elastin in the extracellular matrix (ECM) and contributing to the tensile strength and structural integrity of many tissues^2^. In addition to the commonly known function of ECM crosslinking, more recent emerging roles independent of secretion have been attributed to several LOX proteins. These novel roles have been associated with their intracellular and intranuclear localization^3^. Moreover, amine oxidase catalytic activity is not always required for some of the recently reported LOX functions^4^, suggesting complex and wide-ranging roles for members of the LOX family.

The expression of LOX family proteins is tightly controlled during normal development; however, abnormal expression and activity of these proteins have been reported in a number of diseases^5–8^, particularly in cancers^9–12^. Interestingly, both upregulation and downregulation of LOX family members have been associated with human diseases. For instance, significant decreases in *LOXL2* expression were observed in ovarian tumors^13^ and non-small-cell lung cancer tissues^14^. There is also evidence that high *LOXL2* expression correlates with tumor metastasis^15–17^. These conflicting results are possibly due to the multiple temporal and spatial expression patterns of LOX family members, which may confer differential functions.

Preeclampsia is a pregnancy-specific disorder characterized by hypertension and proteinuria that occurs 20 weeks after gestation^18,19^. Preeclampsia is a major cause of maternal and fetal morbidity and mortality with a prevalence of 6–8% of pregnancies^20^. The pathophysiological mechanism of preeclampsia has not been elucidated; however, it is well known that preeclampsia is associated with impaired trophoblast invasion in early pregnancy^18^, which is responsible for the subsequent oxidative stress and angiogenic imbalance that contributes to endothelial dysfunction during later gestation periods in preeclampsia patients^21,22^. Consequently, increased efforts to investigate the molecular mechanisms controlling trophoblast cell invasion would be helpful for understanding the pathogenesis of preeclampsia.

The sequence of events leading to trophoblast invasion includes cellular attachment to the host tissue, transmigration through the basal lamina, stromal infiltration, and aggressive penetration into blood vessels, which is similar to tumor cell invasion^23^. Considering the complex roles of LOX proteins in tumor cell invasion, we speculated that members of the LOX family are potentially involved in preeclampsia pathogenesis by interfering with the biological behavior of trophoblasts. Therefore, to test our hypothesis that altered expression of LOX family members may result in impaired trophoblast functions in preeclampsia, this study aimed to determine the differential expression of LOX family members between normal pregnancies and preeclampsia patients, evaluate the effects of LOX proteins on trophoblast cell behaviors, and reveal the molecular mechanisms of LOX proteins regulating trophoblast cell behaviors.

## Materials and methods

### Human sample collection

Placental tissues were obtained immediately (<30 min) from normal pregnant women and preeclampsia patients after delivery by cesarean section at Shanghai First Maternity and Infant Hospital. The clinical characteristics of the subjects are summarized in Table S1. The preeclampsia group was defined as onset of hypertension 20 weeks after gestation with a systolic blood pressure of 140 mmHg and/or diastolic blood pressure of 90 mmHg at least two separate measurements (at least 4 h, but with a ≤7-day interval) and consistent proteinuria (300 mg in a 24-h urine collection period or 1+ protein by dipstick detection) according to the guidelines of the US National Institutes of Health^24^. Small pieces (approximately 0.5 cm^3^) were cut from the fetal part of the placenta under aseptic conditions and washed with sterile phosphate-buffered saline (PBS). Chorionic villous samples in the first trimester of pregnancy were randomly collected from women who underwent legal termination for nonmedical reasons of an apparently normal early pregnancy (7–10 weeks’ gestation) at the same hospital during the same period. None of these subjects had a history of spontaneous abortion, ectopic pregnancy, preterm delivery, or stillbirth. Chorionic villous tissues were dissected immediately after vacuum aspiration and washed with sterile PBS. The dissected tissues were immediately snap-frozen and stored in liquid nitrogen until protein, RNA, or collagen preparation. The remaining tissues were fixed at 4 °C using 4% paraformaldehyde and embedded in paraffin for histological analysis. This study was approved by the Scientific and Ethical Committee of the Shanghai First Maternity and Infant Hospital affiliated with Tongji University. All study participants provided written informed consent.

### Histological analysis

Protein expression of LOX family members was detected using immunohistochemistry assays with antibodies against LOX (NB100-2527, 1:300, Novus Biologicals, Littleton, CO, USA), LOXL1 (sc-66949, 1:100, Santa Cruz Biotechnology, Dallas, TX, USA), LOXL2 (sc-48723, 1:100, Santa Cruz Biotechnology), LOXL3 (37906, 1:300, US Biological, Swampscott, MA, USA), and LOXL4 (ALX-215-067-R050, 1:100, Enzo Life Sciences, Farmingdale, NY, USA). Collagen expression was evaluated by Masson’s trichrome staining. The experiments were repeated with at least three different samples from each group. Image acquisition was performed using a Pannoramic 250 Flash digital microscope (3DHISTECH, Budapest, Hungary).

### Cell culture

The HTR-8/SVneo cell line used in this study was a kind gift from Dr. C.H. Graham at Queen’s University, Canada^25^. Cell line authentication was performed using short tandem repeat markers. HTR-8/SVneo cells were cultured in Dulbecco’s modified Eagle medium containing Nutrient Mixture F-12 media (Gibco, Life Technologies, Grand Island, NY, USA) supplemented with 10% fetal bovine serum (FBS; Gibco) at 37 °C under 5% CO_2_ humidified air.

### Immunofluorescence microscopy

HTR-8/SVneo cells were seeded in glass-bottom cell culture dishes and grown to 70% confluence. Then, the cells were fixed with 4% paraformaldehyde in PBS for 20 min and permeabilized with 0.1% Triton X-100 in PBS for 10 min at room temperature. Following washing in PBS and blocking by incubation in 5% (w/v) bovine serum albumin (BSA) in PBS (buffer A), cells were incubated overnight with anti-LOX (ab174316, 1:100, Abcam, Cambridge, MA, USA) and anti-LOXL2 (ab96233, 1:100, Abcam) antibodies diluted in buffer A. Following washing, the cells were incubated for 1 h in Alexa Fluor 488 goat anti-rabbit secondary antibodies (A11070, 1:200 for LOX and 1:1000 for LOXL2, Invitrogen, Carlsbad, CA, USA) diluted in buffer A. The nuclei were stained with 4′,6-diamidino-2-phenylindole. Fluorescence images were obtained using confocal microscopy (TCS SP8; Leica,Wetzlar, Germany).

### Small interfering RNA assays

Small interfering RNA (siRNA; Genepharma, Shanghai, China) was used to investigate the effects *LOX* and *LOXL2* exerted on the biological functions of HTR-8/SVneo cells. The siRNA sequences were as follows: LOX-S1 sense, 5′-GUAAUUACAGAAUUGAAACACUGUGUU-3′; LOX-S2 sense, 5′-ACAGGGAUUGAGUCCUGGCUGUUAU-3′; LOXL2-S1 sense, 5′-GAAGGAGACAUCCAGAAGATT-3′; LOXL2-S2 sense, 5′-CACAUAGGUGGUUCCUUCATT-3′; and negative control sense, 5′-UUCUCCGAACGUGUCACGUTT-3′. One day prior to transfection, HTR-8/SVneo cells were seeded to obtain 30–50% confluency the next day. Cells were transfected with 50 nmol/L of siRNA using Lipofectamine RNAiMAX transfection reagent (Invitrogen) in Opti-MEM I reduced serum medium (Invitrogen) according to the manufacturer’s instructions. Nontargeting siRNA (50 nmol/L) was used as the negative control. One day after transfection (24 h), the cells were collected for migration and invasion assays or incubated for another 24 h with or without the Smad3 inhibitor SIS3 (10 μmol/L; Selleck, Houston, TX, USA) before harvesting.

### Cell proliferation assays

HTR-8/SVneo cells were seeded in 96-well plates at a density of 2000 cells/well. The next day, the attached cells were treated with siRNA. The cell numbers were determined at 24, 48, and 72 h after treatment using a [3-(4,5-dimethylthiazol-2-yl)-5-(3-carboxymethoxyphenyl)-2-(4-sulfophenyl)-2H-tetrazolium, inner salt; MTS]-based cell proliferation assay kit (Promega, Madison, WI, USA) according to the manufacturer’s instructions.

### In vitro migration/invasion assays

Cell migration and invasion were evaluated using a 24-multiwell BD Falcon FluoroBlok Insert System (8.0-μm pores; BD Biosciences, San Jose, CA, USA). The Transwell inserts were coated with Matrigel (BD Biosciences) for the invasion assay. After treatment with siRNA or transforming growth factor-β1 (TGF-β1) for 24 h, HTR-8/SVneo cells were seeded into the insert (topside of the membrane) in media supplemented with 1% FBS at a density of 5 × 10^4^ cells/well for the migration assay and 10 × 10^4^ cells/well for the invasion assay. The bottom wells of the chamber were filled with media supplemented with 10% FBS. After 16 h for the migration assay or 24 h for the invasion assay, cells that had migrated to the underside of the inserts were stained with calcein AM (Invitrogen) for 30 min and then imaged by an inverted microscope mounted with a charge-coupled device camera. The number of migrated cells was counted using the ImageJ software (NIH, Bethesda, MD, USA).

### RNA extraction and quantitative reverse-transcription PCR

Total RNA was isolated using the TRIzol reagent (Sigma-Aldrich, St. Louis, MO, USA). First-strand cDNA was synthesized from 500 ng total RNA with a high-capacity cDNA reverse-transcription kit (Takara, Otsu, Shiga, Japan). Quantitative reverse-transcription PCR (qPCR) was performed using a StepOne analyzer (Applied Biosystems, Carlsbad, CA, USA) with SYBR Green dye (Tiangen Biotech, Beijing, China) according to the manufacturer’s protocol. Relative quantification was performed by means of the 2^−ΔCT^ or 2^−ΔΔCT^ method. *ACTB* and *GAPDH* were chosen as reference genes. The qPCR primers used are listed in Table S2.

### Enzyme-linked immunosorbent assay

The concentration of total TGF-β1 and active TGF-β1 in the HTR-8/SVneo cell culture supernatant was measured using TGF-β1 enzyme-linked immunosorbent assay (ELISA) kits (Biolegend, San Diego, CA, USA) according to the manufacturer’s instructions.

### Western blot analysis

Placental tissue and HTR-8/SVneo cell lysates were prepared using RIPA lysis buffer (Beyotime Biotechnology, Haimen, China) supplemented with a protease inhibitor cocktail and phosphatase inhibitor cocktail (Roche, Branford, CT, USA). Proteins were separated by SDS-polyacrylamide gel electrophoresis and transferred to a polyvinylidene fluoride (PVDF) membrane (Millipore, Billerica, MA, USA). The membrane was blocked with 7% nonfat milk or 7% BSA for 1–3 h at room temperature and incubated with antibodies against LOX (NB100-2527, 1:2000, Novus Biologicals), LOXL1 (sc-66949, 1:500, Santa Cruz Biotechnology), LOXL2 (sc-48723, 1:1500, Santa Cruz Biotechnology), LOXL3 (37906, 1:500, US Biological), LOXL4 (ALX-215-067-R050, 1:1000, Enzo Life Sciences), Phospho-Smad2/3 (5678s, 1:1000, Cell Signaling Technology, Danvers, MA, USA), Smad2/3 (8828s, 1:1000, Cell Signaling Technology), type I collagen (AB758, 1:750, Millipore), type III collagen (ab7778, 1:1000, Abcam), type IV collagen (ab6586, 1:1000, Abcam), β-actin (12262S, 1:4000, CST), GAPDH (51332S, 1:2000, CST), and tubulin (12351S, 1:2000, CST) at 4 °C overnight. PVDF membrane-bound antibodies were detected with horseradish peroxidase-conjugated horse anti-rabbit (M21002S, 1:5000, Abmart), anti-mouse (M21001S, 1:3000, Abmart), or anti-goat IgG (SH-0131, 1:3000, Dingguo Changsheng Biotech CO. LTD, Beijing, China) for 1.5 h at room temperature and then visualized using an enhanced chemiluminescence solution (Millipore). Relative protein expression levels were analyzed by densitometry using the ImageJ software.

### Collagen measurements

Total soluble collagen from cell culture supernatants was quantified using a sircol collagen assay according to the manufacturer’s instructions (Biocolor, Belfast, UK). Briefly, cell culture supernatants were collected and concentrated overnight at 4 °C. Sirius red dye was added to the concentrated collagen solution. The resulting solution was incubated at room temperature for 30 min under gentle rotation. After centrifugation at 12,000 rpm for 10 min, the collagen-bound dye was washed and redissolved. The absorbance was then measured at 555 nm, as it is directly proportional to the amount of collagen present in the cell culture. To determine the total placenta collagen content, we measured the content of hydroxyproline, a major collagen component, as previously described^26^. Placentas were hydrolyzed in hydrochloric acid and then hydroxyproline levels were measured by a colorimetric method using an assay kit (QuickZyme Biosciences, Burlington, NC, USA) according to the manufacturer’s instructions. The total collagen content was calculated from the hydroxyproline content of collagen standards.

### Statistical analysis

The data are presented as the means ± SEM. Statistical differences were analyzed by Student’s *t*-test or one-way analysis of variance using GraphPad Prism version 6 (GraphPad software, San Diego, CA, USA). If unpaired data did not have similar variances, the *t*-test with Welch’s correction was performed. Data were considered significant at *P* < 0.05.

## Results

### *LOX* and *LOXL2* were downregulated in preeclamptic placentas

**Fig. 1:**
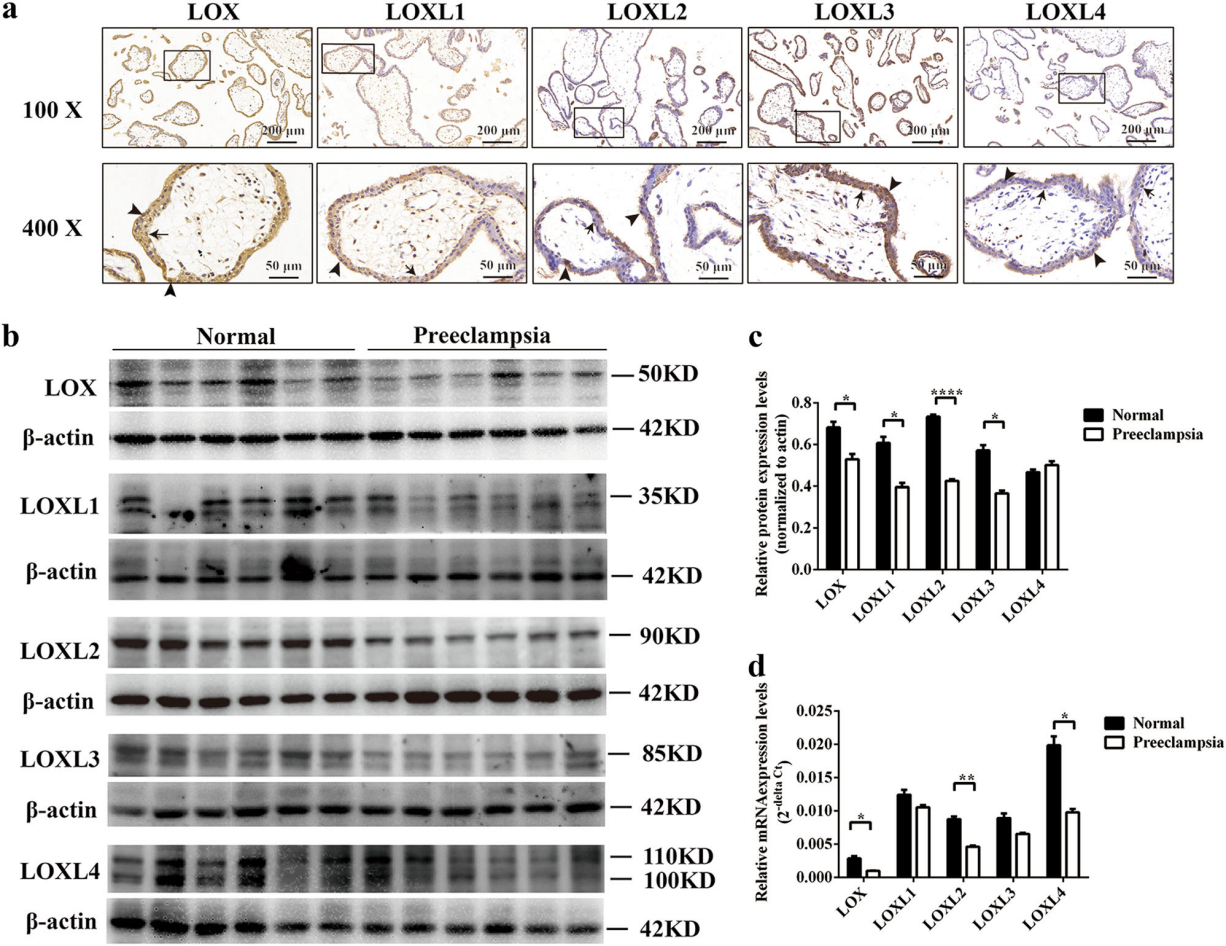
Decreased expression of *LOX* and *LOXL2* was found in preeclamptic placentas. **a** Representative images of expression and localization of LOX family members in first trimester villi by immunohistochemistry analysis. Brownish color represents positive staining of LOX family members. Arrows, trophoblasts; arrowheads, cytotrophoblasts. **b** Western blot analysis of LOX family members in placentas from normal full-term pregnancies and preeclampsia patients. **c** Statistical analysis of protein densitometry quantification of western blot analysis (**b**) by Student’s *t*-test. Data are presented as the means ± SEM. **d** mRNA expression of LOX family members was analyzed by quantitative reverse-transcription PCR. Statistical data were analyzed by Student’s *t*-test. Data are presented as the means ± SEM. *P < 0.05; **P < 0.01; and ****P < 0.0001

To dissect the underlying function of LOX proteins in the placenta, we first performed immunohistochemistry to detect the localization of LOX proteins in first trimester villi. It was observed that LOX, LOXL1, LOXL2, LOXL3, and LOXL4 were mainly localized to the cytoplasm of syncytiotrophoblasts and cytotrophoblasts. Positive LOX protein signals were also detected within the nucleus (Fig. 1a). Additionally, we analyzed the expression levels of LOX family members in the placentas of full-term normal pregnancies and preeclamptic patients using Western blotting and qPCR. Protein levels of LOX, LOXL1, LOXL2, and LOXL3 as well as mRNA levels of *LOX*, *LOXL2*, and *LOXL4* were significantly decreased in preeclamptic placentas (Fig. 1b–d). These results revealed that only *LOX* and *LOXL2* had simultaneous abnormal protein and mRNA expression in preeclamptic placentas.

### *LOX* or *LOXL2* downregulation suppressed trophoblast cell migration and invasion

**Fig. 2:**
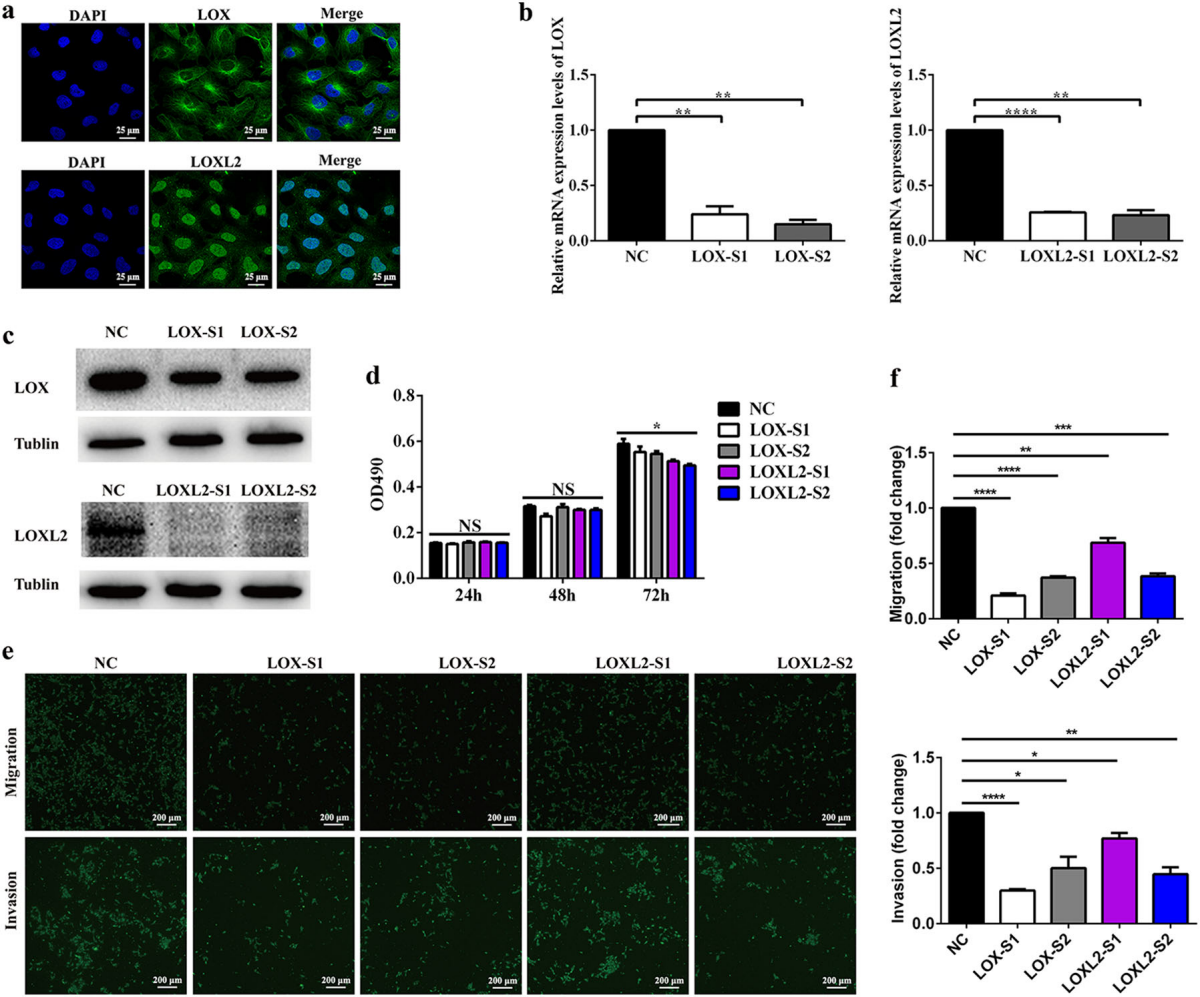
Knockdown of *LOX* or *LOXL2* decreased trophoblast cell migration and invasion. **a** Immunofluorescence staining for LOX and LOXL2 in HTR-8/SVneo cells. Confocal microscopy images were shown. Scale bar = 25 μm. **b** mRNA expression of *LOX* and *LOXL2* in HTR-8/SVneo cells transfected with *LOX* siRNA, *LOXL2* siRNA, or negative control siRNA was analyzed by quantitative reverse-transcription PCR. **c** Western blot analysis of LOX and LOXL2 protein expression in HTR-8/SVneo cells transfected with *LOX* siRNA, *LOXL2* siRNA, or negative control siRNA. **d** Proliferation of HTR-8/SVneo cells lines detected by MTS assays. Statistical analysis of cell proliferation index was performed by one-way analysis of variance. **e** HTR-8/SVneo cell migration and invasion were determined by Transwell assays. Representative images are shown. **f** Relative fold changes in cell migration and invasion were calculated. Statistical data were analyzed by Student’s *t*-test. All data are presented as the means ± SEM of five independent experiments. *P < 0.05; **P < 0.01; ***P < 0.001; ****P < 0.0001; and NS not significant

Considering the localization of LOX proteins in trophoblasts from first-trimester villi and the downregulation of *LOX* and *LOXL2* in preeclamptic placentas, we speculated that loss of function of *LOX* or *LOXL2* in trophoblast cells is critical for preeclampsia. To test this hypothesis, we attempted to investigate the roles of *LOX* and *LOXL2* in the human trophoblast cell line HTR-8/SVneo by reducing *LOX* or *LOXL2* expression using siRNA. First, the subcellular location of LOX and LOXL2 in HTR-8/SVneo cells was detected using immunofluorescence microscopy. LOX was predominantly detected in the perinuclear region/cytoplasmic region and LOXL2 was localized in the nucleus (Fig. 2a). Then, the knockdown effects of siRNA on *LOX* and *LOXL2* expression were verified by qPCR and western blotting (Fig. 2b, c). We next performed cell proliferation assays. The representative results showed that small amplitude decreases in cell number were observed in the knockdown groups 72 h after siRNA treatment, implying that downregulation of *LOX* or *LOXL2* had minor effects on trophoblast cell proliferation (Fig. 2d). Furthermore, HTR-8/SVneo cell migration and invasion were assessed by Transwell assays. The results showed that decreased *LOX* or *LOXL2* expression suppressed HTR-8/SVneo cell migration and invasion (Fig. 2e, f). Taken together, these results suggest that *LOX* and *LOXL2* are required for trophoblast cell migration and invasion.

### Collagen production was induced in *LOX*- or *LOXL2*-downregulated preeclamptic placenta and trophoblast cells

**Fig. 3:**
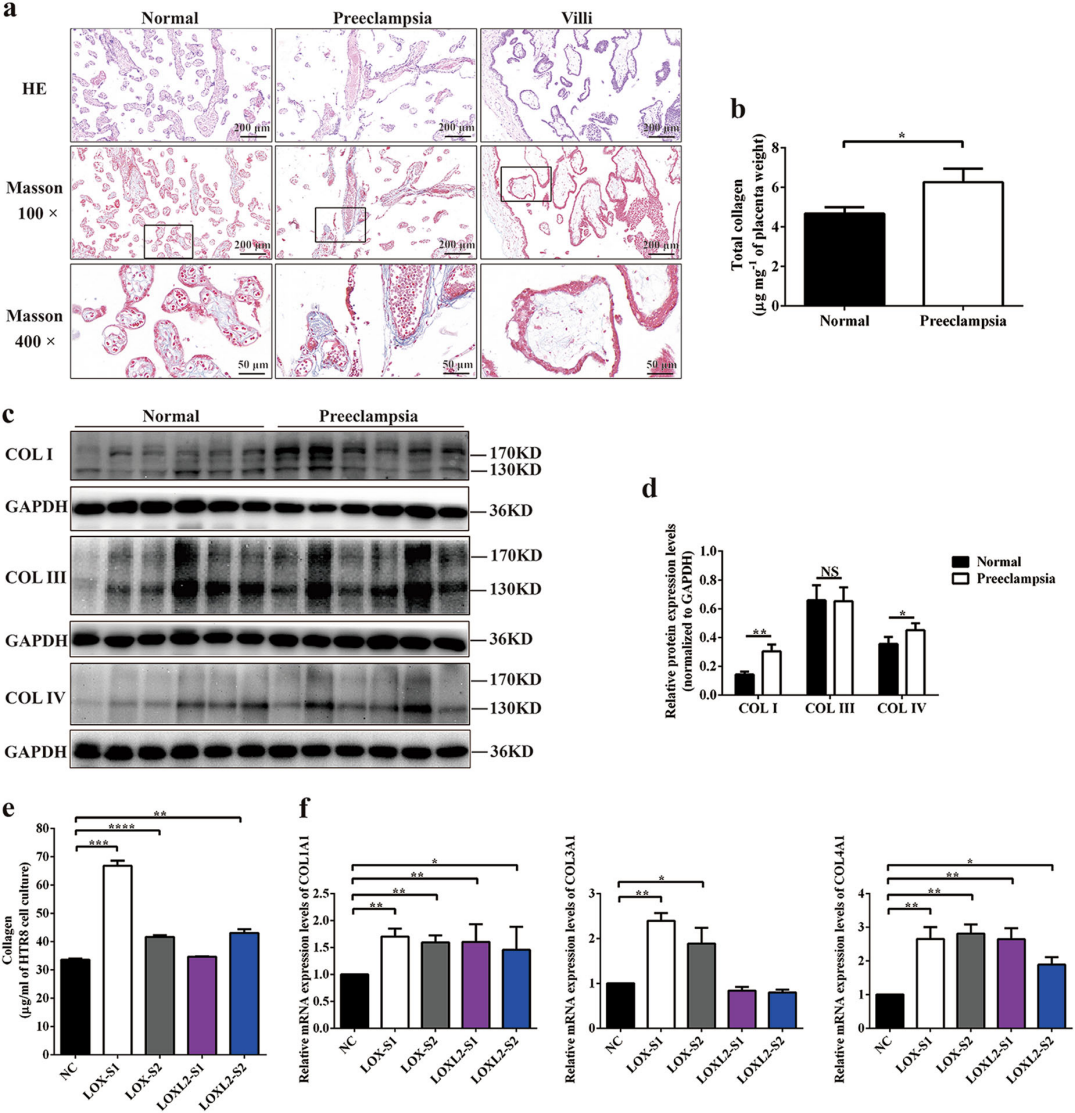
Collagen production was induced in *LOX*- or *LOXL2*-downregulated preeclamptic placenta and trophoblast cells. **a** Representative hematoxylin-eosin staining and Masson’s trichrome staining of placenta and villi are shown. **b** Amount of total collagen was determined by measuring the hydroxyproline contents of placental tissues. **c** Western blot analysis of type I collagen, type III collagen, and type IV collagen in the placenta. **d** Statistical analysis of protein densitometry quantification of western blot analysis (**c**) by Student’s *t*-test. Data are presented as the means ± SEM. **e** Amount of soluble collagen from cell culture supernatants was quantified using a sircol collagen assay. Data are presented as the means ± SEM of three independent experiments. **f** Quantification of *COL1A1*, *COL3A1*, and *COL4A1* mRNA expression levels determined in HTR-8/SVneo cells after siRNA treatment. Data are presented as the means ± SEM of three independent experiments. *P < 0.05; **P < 0.01; ***P < 0.001; ***P < 0.001; and NS not significant

Considering that LOX proteins are crucial for collagen fiber crosslinking, we theorized that downregulation of *LOX* or *LOXL2* in trophoblast cells would change the structural organization of collagen in the ECM. To validate this hypothesis, we evaluated the distribution of collagen in first-trimester villi and full-term placentas using Masson’s trichrome staining. The results revealed that collagen was richer in preeclamptic placentas than in normal full-term placentas or first-trimester villi (Fig. 3a). For more quantitative results, total amounts of collagen were determined by measuring the hydroxyproline contents in the placentas; the collagen content was found to be significantly increased in preeclamptic placentas compared with normal full-term placentas (Fig. 3b). Additionally, elevated production of type I and type IV collagen was observed in preeclamptic placentas by western blotting (Fig. 3c, d). These results indicate that collagen production is induced in placentas from preeclampsia patients.

To further verify whether collagen expression is regulated by LOX and/or LOXL2, we examined the collagen contents after *LOX* or *LOXL2* knockdown in HTR-8/SVneo cells. The results showed that knockdown of *LOX* or *LOXL2* resulted in a significant increase in soluble collagen contents in the cell culture supernatant (up to twofold higher in the LOX-S1 knockdown group; Fig. 3e). Furthermore, transcription levels of *COL1A1*, *COL3A1*, and *COL4A1* were determined by qPCR; we found that the mRNA levels of *COL1A1* and *COL4A1* were significantly enhanced in HTR-8/SVneo cells with decreased *LOX* or *LOXL2* expression (Fig. 3f). Collectively, these results indicate that collagen expression is controlled by *LOX* and *LOXL2* in trophoblast cells.

### TGF-β1/Smad3 signaling was activated in *LOX*- or *LOXL2*-downregulated trophoblast cells and preeclampsia placentas

**Fig. 4:**
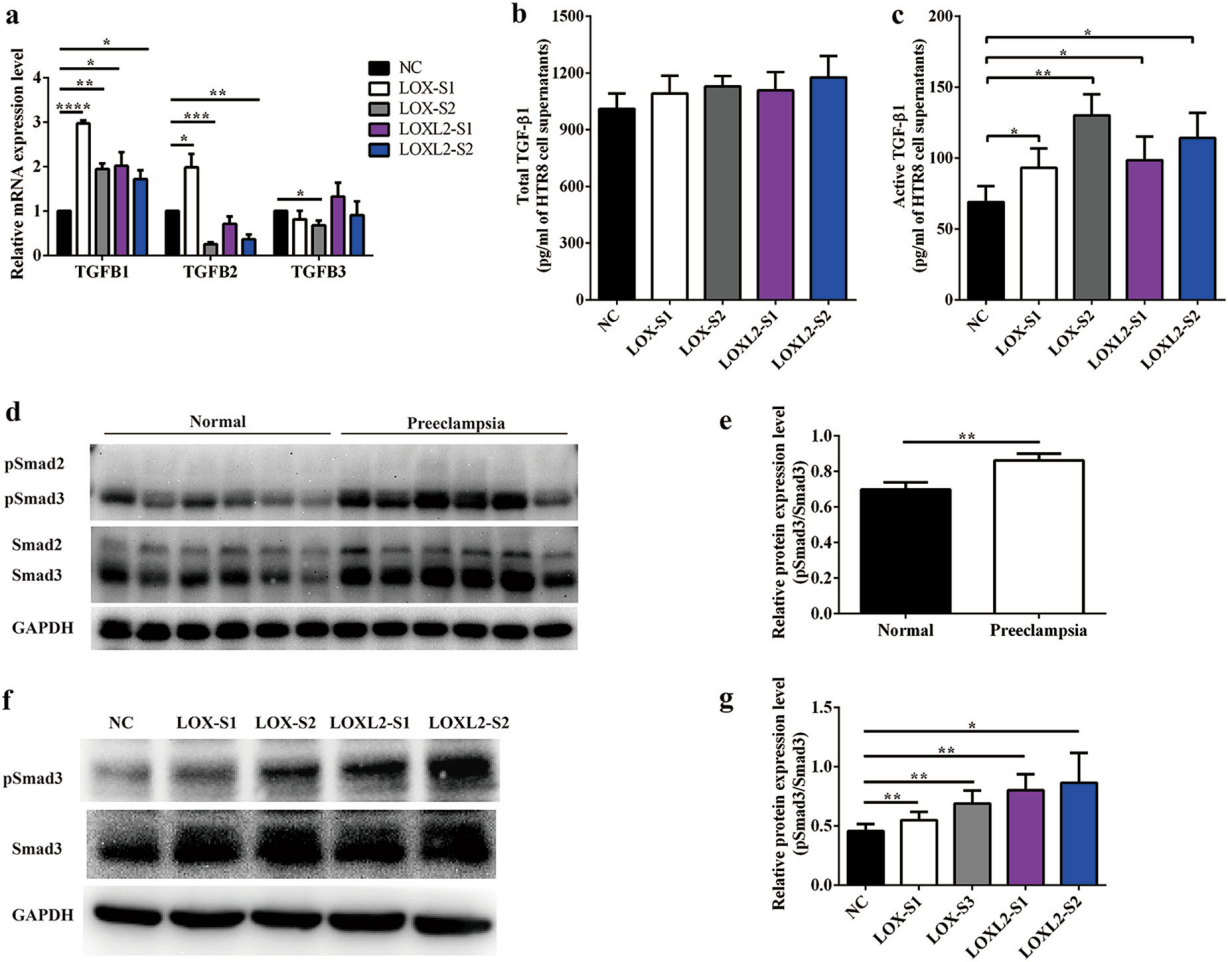
TGF-β1/Smad3 signaling was activated in *LOX*- or *LOXL2*-downregulated trophoblast cells. **a** mRNA expression of *TGFB1*, *TGFB2*, and *TGFB3* was determined by quantitative reverse-transcription PCR. **b**, **c** Total and active transforming growth factor-β1 (TGF-β1) protein levels in culture supernatants from HTR-8/SVneo cells transfected with small interfering RNA (siRNA) were measured by enyme-linked immunosorbent assay. **d** Western blotting and **e** quantification analysis of pSmad2/3 and Smad2/3 in placentas from normal pregnant women and preeclampsia patients. **f** Western blotting and **g** quantification analysis of pSmad2/3 and Smad2/3 in HTR-8/SVneo cells transfected with siRNA. Statistical data were analyzed by Student’s *t*-test. Data are presented as the means ± SEM of three independent experiments. *P < 0.05; **P < 0.01; ***P < 0.001; and ****P < 0.0001

TGF-β has been reported to stimulate ECM production and collagen architecture alterations. These findings prompted us to explore whether *LOX* and/or *LOXL2* modulate(s) collagen expression in trophoblast cells through the TGF-β pathway. We first examined the mRNA transcription levels of *TGFB1*, *TGFB2*, and *TGFB3* after *LOX* or *LOXL2* knockdown in HTR-8/SVneo cells. The results showed that mRNA expression levels of *TGFB1* were significantly increased in the *LOX* or *LOXL2* knockdown groups (Fig. 4a). Next, we performed ELISA to analyze TGF-β1 levels. We found that active TGF-β1 but not total TGF-β1 was induced after *LOX* or *LOXL2* downregulation in HTR-8/SVneo cells (Fig. 4b, c). Additionally, the mRNA and protein levels of *TGFB1* in placenta tissues were detected. The results showed that the mRNA expression of *TGFB1* (Figure S1-a) and active TGF-β1 protein levels (Figure S1-b) were increased in the placental tissues from preeclampsia patients compared with normal control groups.

Smad2/3 proteins are considered important signal transducers in the TGF-β1-inducing signaling pathway; phosphorylated Smad2/3 proteins can bind to Smad4 and translocate to the nucleus where they bind and activate promoters with Smad-binding elements^27^. Therefore, phosphorylation levels of Smad2/3 were evaluated by western blotting in clinical samples and HTR-8/SVneo cells upon siRNA treatment. Compared with normal full-term placentas, phosphorylation levels of Smad3 were significantly upregulated in preeclamptic placentas with lower *LOX* and *LOXL2* expression (Fig. 4d, e). Consistently, reducing *LOX* or *LOXL2* expression in HTR-8/SVneo cells by siRNA treatment led to an increase in phosphorylated Smad3 protein levels (Fig. 4f, g). Taken together, these results suggest that *LOX* or *LOXL2* downregulation activates the TGF-β1/Smad3 signaling pathway.

### Inhibiting Smad3 could partially rescue trophoblast cell migration and invasion

**Fig. 5:**
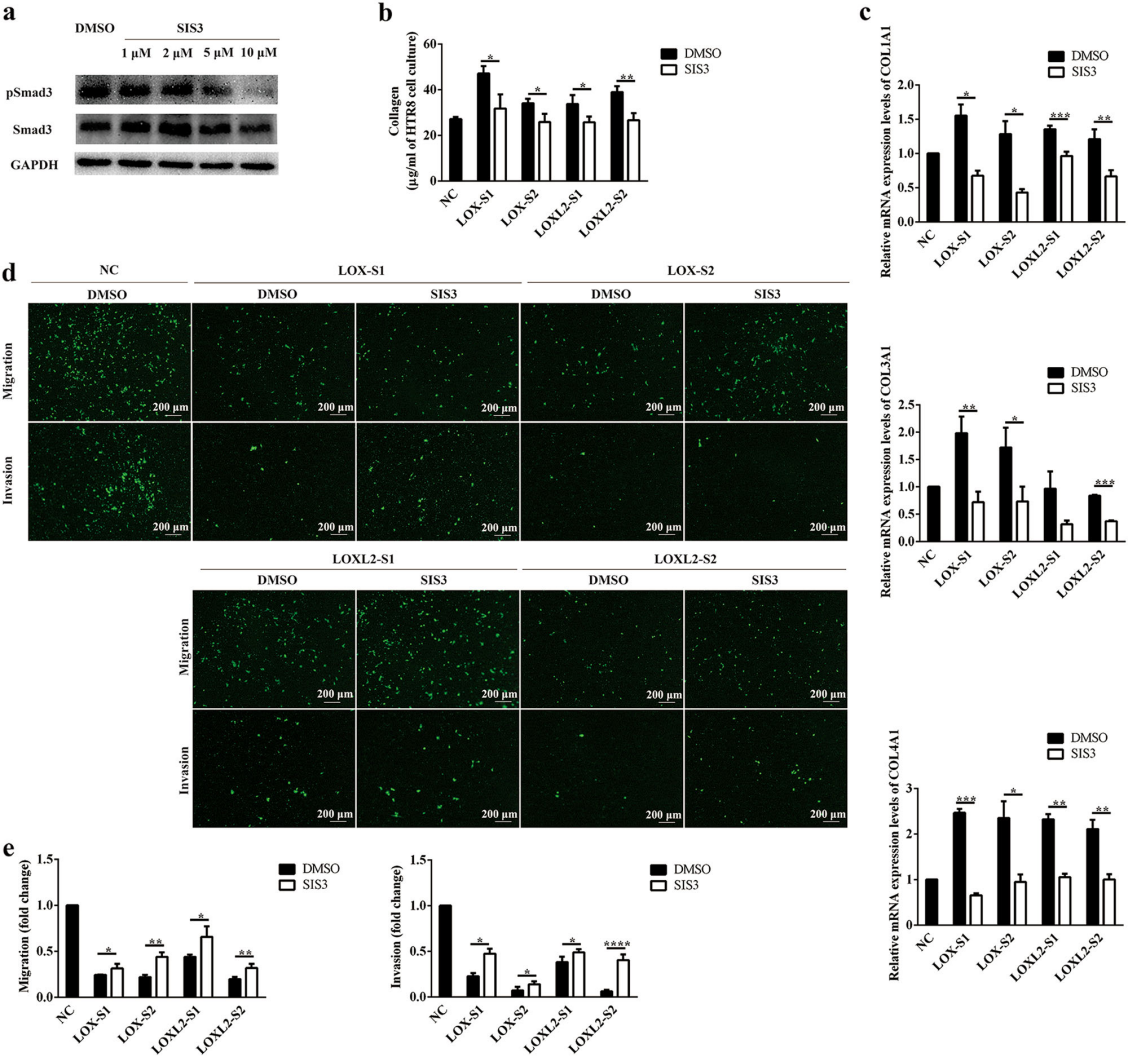
Inhibiting Smad3 could partially rescue trophoblast cell migration and invasion. **a** Western blot analysis of pSmad3 and Smad3 in HTR-8/SVneo cells treated with the Smad3 inhibitor SIS3 (0, 2, 5, or 10 μmol/L) for 24 h. **b** Amount of soluble collagen in culture supernatants from HTR-8/SVneo cells transfected with small interfering RNA (siRNA) followed by SIS3 treatment (10 μmol/L) was quantified using a sircol collagen assay. **c** Quantification of *COL1A1*, *COL3A1*, and *COL4A1* mRNA expression levels was determined in HTR-8/SVneo cells upon siRNA transfection followed by SIS3 treatment (10 μmol/L). **d** Migration and invasion of HTR-8/SVneo cells upon siRNA transfection followed by SIS3 treatment (10 μmol/L) was determined by Transwell assays. Representative images are shown. **e** Relative fold changes in cell migration and invasion were calculated. Statistical data were analyzed by Student’s *t*-test. Data are presented as the means ± SEM of three independent experiments. *P < 0.05; **P < 0.01; ***P < 0.001; and ****P < 0.0001

To understand the importance of TGF-β1 signaling as a regulator downstream of LOX and LOXL2, we performed rescue experiments in trophoblast cells. For this purpose, *LOX*- or *LOXL2*-downregulated HTR-8/SVneo cells were treated with the Smad3 inhibitor SIS3. Then, collagen expression levels and cell migration and invasion abilities were examined. The inhibitory effects of SIS3 on Smad3 phosphorylation levels were confirmed by western blot analysis (Fig. 5a). Reduced Smad3 phosphorylation reversed the inducing effects of *LOX* or *LOXL2* knockdown on collagen production (Fig. 5b, c) and partially reversed the inhibitory effects of *LOX* or *LOXL2* knockdown on trophoblast cell migration and invasion (Fig. 5d, e). These results imply that Smad3 signaling is an important downstream factor of *LOX* and *LOXL2* that modulates trophoblast cell migration and invasion.

### Exogenous TGF-β1 treatment reduced trophoblast cell invasion

**Fig. 6:**
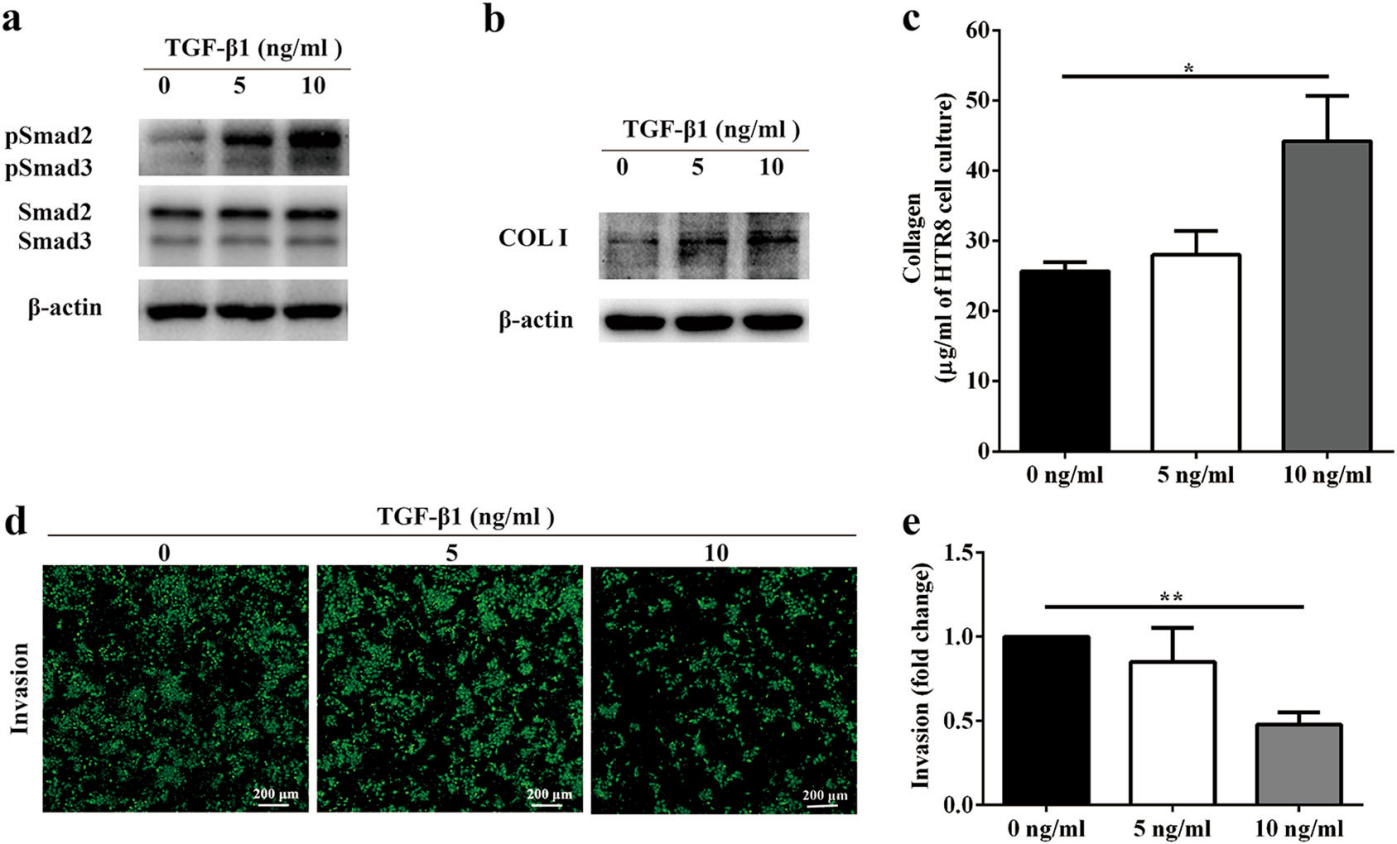
Exogenous transforming growth factor-β1 (TGF-β1) treatment reduced trophoblast cell invasion. **a** Western blot analysis of pSmad3 and Smad3 in HTR-8/SVneo cells stimulated by exogenous TGF-β1. **b** Western blot analysis of type I collagen production in HTR-8/SVneo cells stimulated by exogenous TGF-β1. **c** Amount of soluble collagen from cell culture supernatants was quantified using a sircol collagen assay. Data are presented as the means ± SEM of three independent experiments. **d** Invasion of HTR-8/SVneo cells upon TGF-β1 treatment was determined by Transwell assays. Representative images are shown. **e** Relative fold changes in cell invasion were calculated. Statistical data were analyzed by Student’s *t*-test. Data are presented as the means ± SEM of three independent experiments. *P < 0.05 and **P < 0.01

To further verify the importance of the TGF-β1/Smad3/collagen pathway as a regulator of trophoblast cell movement, we performed invasion assays in HTR-8/SVneo cells. For this purpose, exogenous TGF-β1 was used to stimulate HTR-8/SVneo cells. It was indicated that exogenous TGF-β1 activated Smad2/3 (Fig. 6a) and induced an increase in type I collagen production (Fig. 6b). Additionally, sircol assay showed that collagen secretion was highly promoted by 10 ng/mL exogenous TGF-β1 (Fig. 6c). Furthermore, invasion of HTR-8/SVneo cells were assessed by Transwell assays. The results showed that HTR-8/SVneo cell invasion was suppressed after stimulation with 10 ng/mL exogenously applied TGF-β1 (Fig. 6d, e). Taken together, these results suggest that the TGF-β1/Smad3/collagen pathway is required for trophoblast cell invasion.

## Discussion

Preeclampsia is a potentially life-threatening disorder during human pregnancy that remits after delivery, suggesting that the placenta is central in preeclampsia. Impaired migration properties of placental trophoblast cells lead to insufficient invasion of trophoblasts into the maternal decidua, resulting in improper remodeling of spiral arteries; this is thought to be a significant pathomechanism in preeclampsia. In this study, we uncovered that *LOX* and *LOXL2* were indispensable for trophoblast cell migration and invasion. The molecular mechanism was demonstrated by *LOX* and *LOXL2* modulation of collagen expression through the TGF-β1/Smad3 pathway in trophoblasts. Notably, we found decreased expression levels of *LOX* and *LOXL2* in preeclamptic placentas. Our study demonstrated that downregulation of *LOX* and *LOXL2* is relevant to preeclampsia.

Cell invasion plays an essential role in tumorigenesis^28^ and is a fundamental process during embryonic development^29^. A number of studies have reported LOX family member involvement in the migration and invasion of various tumor cells. Global inhibition of LOX family activity by β-aminopropionitrile inhibited trophoblast invasiveness, strongly suggesting roles for LOX family members in trophoblast invasion^30^. However, the effect of selective inhibition of each LOX isoform on trophoblast invasion has not yet been investigated. In the present study, we reduced *LOX* or *LOXL2* expression in trophoblast cells through siRNA and found that knockdown of *LOX* or *LOXL2* inhibited trophoblast cell migration and invasion. In agreement with these results, it was reported that knockdown of *LOX* or *LOXL2* attenuated the invasion capacities of breast cancer cells^31^ and gastric cancer cells^17^.

It is well known that impaired trophoblast invasion in early pregnancy is closely associated with preeclampsia^18^. As mentioned above, trophoblast cell invasion can be inhibited by the decreased expression of LOX proteins; therefore, it was reasonable to believe that LOX protein expression is reduced in placental tissues from preeclampsia patients. Thus, we compared the expression levels of LOX proteins in placental tissues from normal pregnant women and preeclampsia patients. Our results showed that both mRNA and protein expression of *LOX* and *LOXL2* was decreased in placentas from patients with preeclampsia. These results indicate that *LOX* and *LOXL2* may play important roles in placental development and may be associated with the pathophysiology of preeclampsia.

It was demonstrated that trophoblastic plugs obstruct maternal blood flow into the intervillous space in early pregnancy^32^. At approximately the eleventh week of gestation, the plugs become loose and allow for continuous maternal blood flow into the intervillous space^33^. As a result, the local oxygen partial pressure increases from approximately 18 to 60 mm Hg^34^, thus the placenta develops enhanced mechanisms for protection against oxidative damage^35^. However, placental hypoxia prolonged beyond the first trimester, which causes failed transformation of maternal spiral arteries, has been recognized as a probable cause of pregnancy pathologies, such as preeclampsia^33^. Hypoxia-inducible factors (HIFs) are key transcriptional regulatory factors that mediate cellular adaptation to a hypoxia microenvironment. HIFs consist of an oxygen labile α subunit (HIF-α) and a constitutively expressed β-subunit. HIF-α subunits are hydroxylated at conserved proline residues by prolyl hydroxylasesin environments with sufficient oxygen and rapidly degraded by the ubiquitin-proteasome system. While in the hypoxic environment, the activity of prolyl hydroxylases is diminished, whereupon HIF-α subunits become stabilized and dimerize with HIF-β subunit to bind to the hypoxia response elements of a large number of target genes, such as *LOX* and *LOXL2*^36,37^. In this study, we found that the expression of LOX, LOXL2, and HIF-2α proteins was decreased in HTR-8/SVneo cells after prolonged hypoxic (72 h) treatments (Figure S3). These results indicate that reduced expression of HIF-2α under prolonged hypoxia is a probable cause for the downregulation of *LOX* and *LOXL2* in placental tissues from preeclampsia patients.

Trophoblast cell invasion is a highly integrated, multistep process that responds to extracellular stimuli and involves cell adhesion and motility. Invasive cells penetrate the uterine wall in a process that involves ECM degradation and proteolysis. These ECM components include collagen, fibronectin, laminin, vitronectin, trophin, and tastin^38^. It was reported that trophoblast adhesiveness was highest in the presence of type I and IV collagen compared with other matrix proteins^39^, while LOX was described to activate the transcription of collagen in COS-7 cells^40^. Interestingly, we found that type I and IV collagen expression was negatively regulated by *LOX* and *LOXL2* in trophoblasts, indicating that LOX members may have different roles in gene regulation in different cell types. Notably, increased expression of type I and IV collagen was observed in the placenta of preeclampsia patients in this study. In agreement with our results, a previous study reported that levels of the collagen fragment arresten derived from type IV collagen were significantly increased in second- and third-trimester preeclampsia plasma and in third-trimester preeclampsia deciduas^41^. Furthermore, liquid chromatography-tandem mass spectrometry was used to analyze the urine peptidome of women with preeclampsia and the results revealed that *COL1A1* and *COL3A1* may potentially serve as early indicators of preeclampsia^42^. Overall, these findings indicate that collagen accumulation caused by the downregulation of *LOX* and *LOXL2* may be a key factor in suppressing trophoblast cell migration and invasion in preeclampsia.

TGF-β1 signaling is a well-known driver of collagen expression and tissue accumulation^43^. The collagen stabilization-associated enzymes LOX and LOXL2 have also been reported to be enhanced by TGF-β1^44,45^. However, no significant changes of the expression of *LOX* or *LOXL2* in HTR-8/SVneo cells upon exogenous TGF-β1 treatment were found in our study (Figure S2). The conflicting results indicated that the regulation of *LOX* and *LOXL2* gene expression may vary between different cell types. It should be noted that modulation of *TGFB1* expression by LOX and LOXL2 has not been previously reported. In this study, we found that *TGFB1* expression was induced by *LOX* or *LOXL2* knockdown and that the TGF-β1/Smad3 pathway functioned as a critical factor downstream of LOX and LOXL2 in regulating collagen expression and modulating trophoblast cell migration and invasion. Additionally, we detected that the TGF-β1/Smad3 pathway was induced in placentas from preeclampsia patients. This observation is consistent with a previous report showing that higher concentrations of mean serum TGF-β1 levels were observed in preeclampsia patients compared with normal pregnant women (62.73 vs. 47.01 ng/mL, respectively)^46^. These findings substantiated the significance of the TGF-β1/Smad3 pathway in preeclampsia. However, it should be noted that the Smad3 inhibitor SIS3 failed to fully restore the deficiencies caused by *LOX* or *LOXL2* knockdown, implying that LOX and LOXL2 possibly regulate other downstream modulators to influence trophoblast cell migration and invasion. Thus, future research in uncovering direct LOX- and LOXL2-mediated molecules may provide deeper insights into the function of *LOX* and *LOXL2* in preeclampsia.

Our present study indicated that *LOX* and *LOXL2* played important roles in trophoblast cell migration and invasion by altering the TGF-β1/Smad3 pathway to modulate collagen expression. Furthermore, evaluation of clinical samples revealed that downregulation of *LOX* and *LOXL2* and upregulation of collagen were associated with preeclampsia. Thus, our findings suggest that *LOX*, *LOXL2*, and the TGF-β1/Smad3/collagen pathway are potential markers and targets for clinical diagnosis and therapy for preeclampsia.

## Supplementary information

Supplemental Materials
