# Supplementary material for: Downregulation of lysyl oxidase and lysyl oxidase-like protein 2 suppressed the migration and invasion of trophoblasts by activating the TGF-β/collagen pathway in preeclampsia
Source: Exp Mol Med. 2019 Feb 21;51(2):20. doi: 10.1038/s12276-019-0211-9 (PMC6389995; doi:10.1038/s12276-019-0211-9)
Supplement: Supplementary file 1 — Supplemental Materials [file 12276_2019_211_MOESM1_ESM.docx]

**Downregulation of lysyl oxidase and lysyl oxidase-like protein 2 suppressed the migration and invasion of trophoblasts by activating the TGF-β/collagen pathway in preeclampsia**

**Running title:** Low levels of *LOX/LOXL2* related to preeclampsia

Xiang-Hong Xu, Yuanhui Jia, Xinyao Zhou, Dandan Xie, Xiaojie Huang, Linyan Jia, Qian Zhou, Qingliang Zheng, Xiangyu Zhou, Kai Wang^*^, Li-Ping Jin^*^

Clinical and Translational Research Center, Shanghai First Maternity and Infant Hospital, Tongji University School of Medicine, 2699 West Gaoke Road, Shanghai, 201204, P. R. China.

^*^Correspondence to Li-Ping Jin or Kai Wang, Clinical and Translational Research Center, Shanghai First Maternity and Infant Hospital, Tongji University School of Medicine, 2699 West Gaoke Road, Shanghai, 201204, P. R. China. Email: [jinlp01@163.com](mailto:jinlp01@163.com), [kaiwangcn@yahoo.com](mailto:kaiwangcn@yahoo.com). Tel: +86-21-20261228, Fax: +86-21-50730190.

**Supplementary Methods**

**Preparation of placental homogenates and quanlification of TGF-β1 in placental homogenates**

To prepare placental homogenates, placental tissues were washed in ice-cold PBS to remove remaining blood. After this, 60 mg of tissue was grinded in liquid nitrogen and transferred into a plastic tube containing 600 μl ice-cold PBS with complete protease inhibitor cocktail (Sigma-Aldrich, St. Louis, MO, USA). The tissues were fully homogenized with Sonicator 4000 (Qsonica-Misonix, Melville, NY, USA) for 18 seconds on ice. Homogenates were centrifuged at 12000 rpm for 15 min at 4°C. The supernatant was collected and the total protein concentration was determined by BCA Protein Assay Kit (Thermo Fisher Scientific, Waltham, MA, USA). Then the supernatant aliquots were stored at –80 °C until required for enzyme-linked immunosorbent assay quanlification. Concentrations of active TGF-β1 in placental homogenates were determined by enzyme-linked immunosorbent assay kits (Biolegend, San Diego, CA, USA) according to the manufacturer’s instructions. Results were expressed as pg/ng of total proteins of each placental homogenate.

**Hypoxia analysis**

For hypoxic analysis, cells were cultured in 3% O_2_ using a multi-gas incubator (SANYO, Tokyo, Japan) for 24 h, 48 h and 72 h. Control cells were cultured in 21% O_2_.

**Supplementary Tables**

**Table S1: Characteristics of the subjects**

| **Variable** | **Normal**  **(n = 18)** | **Preeclampsia (n = 17)** | **P value^*^** |
| --- | --- | --- | --- |
| Patient age (years ± SEM) | 30.44 ± 0.8174 | 30.12 ± 1.280 | 0.8290 |
| Gestation age (weeks ± SEM) | 39.31 ± 0.15 | 36.87 ± 0.80 | 0.0368 |
| Systolic pressure (mmHg ± SEM) | 115 ± 1.76 | 156 ± 3.32 | ＜0.001 |
| Diastolic pressure (mmHg ± SEM) | 76 ± 1.34 | 101 ± 2.81 | ＜0.001 |
| Proteinuria | NA^$^ | ++~+++ | NA |
| Fetal weight (g ± SEM) | 3423 ± 59.89 | 2942 ± 176.8 | 0.0110 |

^*^ P values were obtained by t test or t test with Welch’s correction using GraphPad Prism software

^$^ NA, not available

**Table S2: Sequences of qPCR primers used in this study**

| **Gene Name** | **Forward Primer** | **Reverse Primer** |
| --- | --- | --- |
| *ACTB* | AGCCTCGCCTTTGCCGAT | CTTCTGACCCATGCCCACC |
| *GAPDH* | GAGTCAACGGATTTGGTCGT | CATGGGTGGAATCATATTGGA |
| *LOX* | AGCATACAGGGCAGATGTCAGAG | CTTGGTCGGCTGGGTAAGAAAT |
| *LOXL1* | TGGCTGAACTCGTCCATGCTGTG | ACTACGATGTGCGGGTGCTACTG |
| *LOXL2* | CACTGCGGATCCCTGAAACC | CTGTCTTCGGGCTGATGATCC |
| *LOXL3* | GGGACCCGCTTCACTGCTGG | GGCCAGGCAGTTCTCTTCCGC |
| *LOXL4* | GATGGGCACCGGGTCTGGCT | CGGCAGCAGCTAGGAGTGTGC |
| *COL1A1* | CATCTGGTGGTGAGACTTGC | TCCTGGTTTCTCCTTTGG |
| *COL3A1* | GTCCCAGCGGTTCTCCA | CCCCGTGCTCCAGTGAT |
| *COL4A1* | ACTCTTTTGTGATGCACACCA | AAGCTGTAAGCGTTTGCGTA |
| *TGFB1* | GCGTGCTAATGGTGGAAAC | CGGTGACATCAAAAGATAACCAC |
| *TGFB2* | CCAAAGGGTACAATGCCAAC | CAGATGCTTCTGGATTTATGGTATT |
| *TGFB3* | GATGATTCCCCCACACCG | CTGCACTGCGGAGGTATG |

**Supplementary Figures**


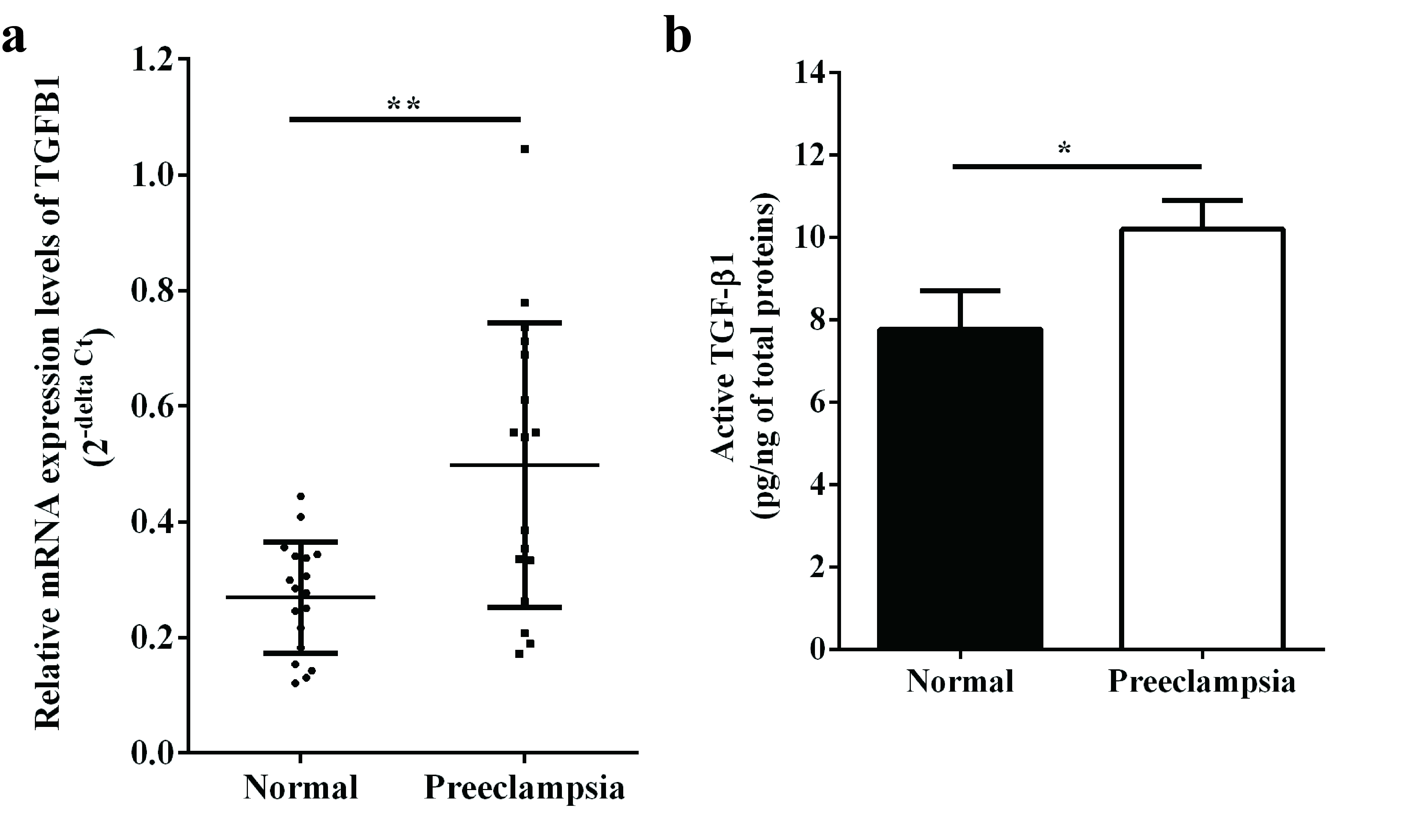


**Figure S1. Increased expression of *TGFB1* was found in preeclamptic placentas.** a, mRNA expression levels of *TGFB1* were analyzed using qPCR. b, Active TGF-β1 protein levels in placentas were measured by enzyme-linked immunosorbent assay. Data are presented as the means ± SEM. **P*<0.05; ***P*<0.01.


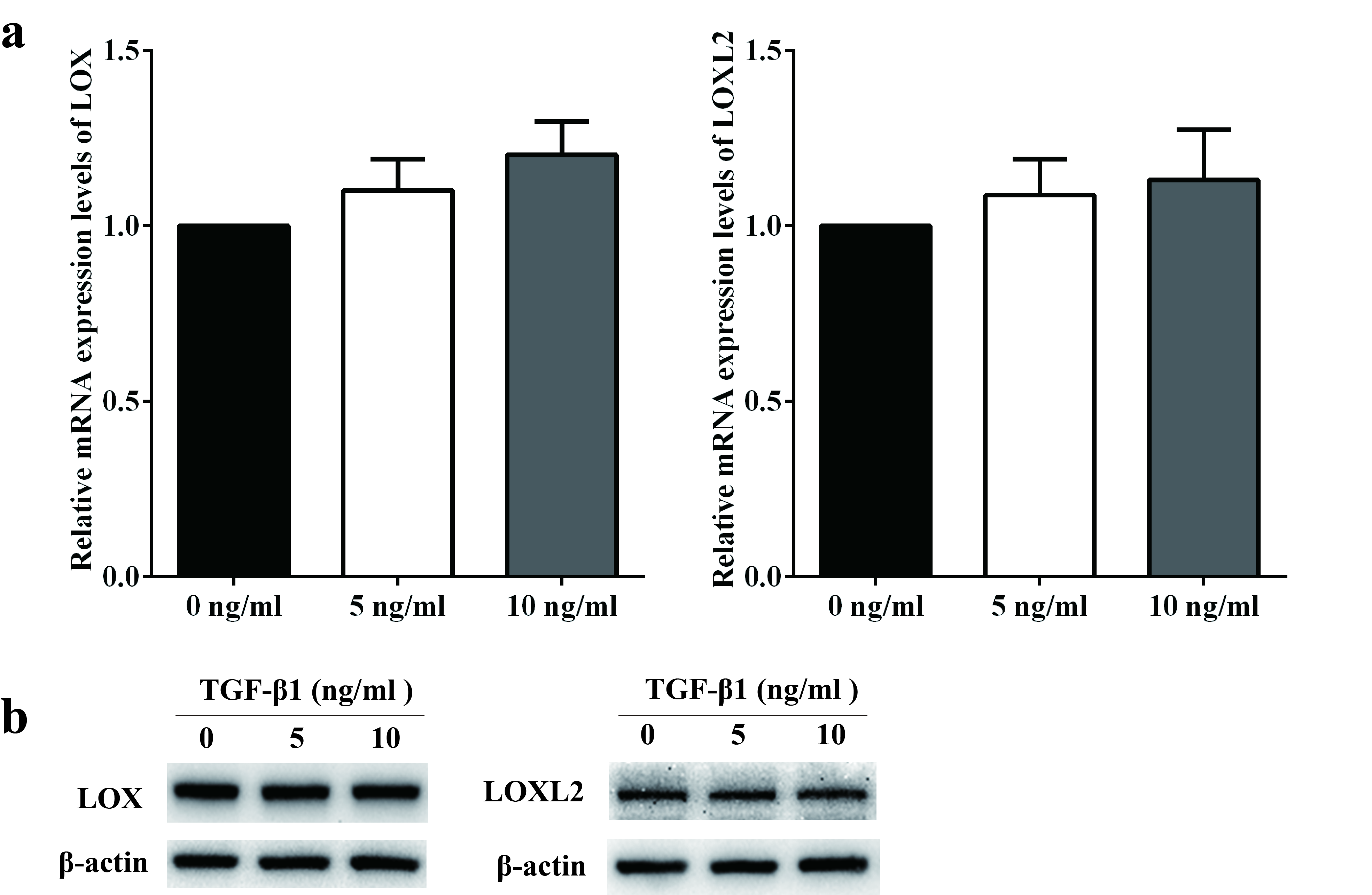


**Figure S2. Expression of *LOX* and *LOXL2* in HTR-8/SVneo cells upon exogenous TGF-β1 treatment**. a, mRNA expression levels of *LOX* and *LOXL2* in HTR-8/SVneo cells treated with TGF-β1 for 48 h were analyzed using qPCR. Data are presented as the means ± SEM of four independent experiments. b, Western blot analysis of LOX and LOXL2 protein levels in HTR-8/SVneo cells treated with TGF-β1 for 48 h.


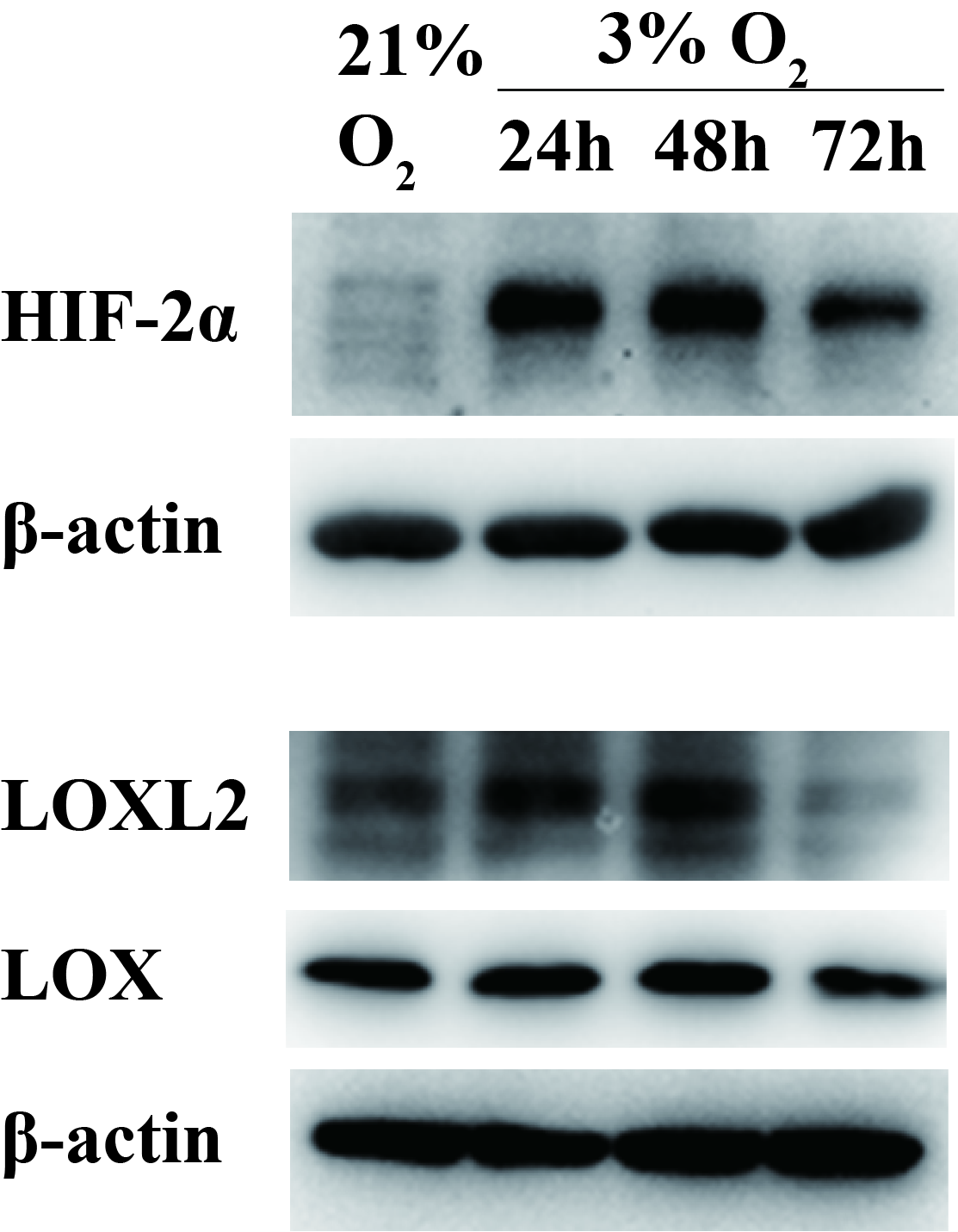


**Figure S3.** Protein expression levels were analyzed by western blot in HTR-8/SVneo cell line under normaxic or 3% O_2_ hypoxic environment. β-actin was used as a loading control.
